# RETRACTION: Involvement of Endoplasmic Reticulum Stress in Capsaicin‐Induced Apoptosis of Human Pancreatic Cancer Cells

**DOI:** 10.1155/ecam/9787656

**Published:** 2026-03-31

**Authors:** Evidence-Based Complementary and Alternative Medicine

RETRACTION: S. Lin, J. Zhang, H. Chen, et al., “Involvement of Endoplasmic Reticulum Stress in Capsaicin‐Induced Apoptosis of Human Pancreatic Cancer Cells,” *Evidence-Based Complementary and Alternative Medicine*, no. 2013 (2013), https://doi.org/10.1155/2013/629750.

The above article, published online on 28 May 2013 in Wiley Online Library (https://wileyonlinelibrary.com), has been retracted by John Wiley & Sons Ltd.

The retraction has been agreed following an investigation of the concerns initially raised by *René Aquarius* on PubPeer [1], which identified multiple instances of inappropriately overlapping figures. Our investigation concluded that there are concerns about the reliability of this article as follows:

- Figure 3a: The dot plots corresponding to PANC‐1 cells treated with 150, 200, and 250 µM of capsaicin appear similar to the dot plots corresponding to 20, 40, and 80 µM emodin in Figure 3 of [2].
- Figure 8a: The β‐tubulin bands share unexpected similarities with the β‐actin bands in Figure 4a of [3]. Similarities were also identified with the β‐actin bands in Figure 5a of [4] after a vertical flip.
- Figure 5c: The PANC‐1 Control and PANC‐1 GADD153 siRNA + Capsaicin dot plots appear similar to the top‐left and top‐right panels of Figure 2a of [5], respectively.

The authors have been informed of the retraction but did not provide a response.
